# Cognitive subtypes of dyslexia are characterized by distinct patterns of grey matter volume

**DOI:** 10.1007/s00429-013-0595-6

**Published:** 2013-06-18

**Authors:** Katarzyna Jednoróg, Natalia Gawron, Artur Marchewka, Stefan Heim, Anna Grabowska

**Affiliations:** 1 Laboratory of Psychophysiology, Department of Neurophysiology, Nencki Institute of Experimental Biology, Pasteur 3, 02-093 Warsaw, Poland; 2 Faculty of Psychology, University of Warsaw, Warsaw, Poland; 3 Laboratory of Brain Imaging, Neurobiology Centre, Nencki Institute of Experimental Biology, Warsaw, Poland; 4 Section Structural-Functional Brain Mapping, Department of Psychiatry, Psychotherapy and Psychosomatics, Medical School, RWTH Aachen University, Aachen, Germany; 5 JARA-Translational Brain Medicine, Jülich and Aachen, Germany; 6 Section Neurological Cognition Research, Department of Neurology, Medical School, RWTH Aachen University, Aachen, Germany; 7 Research Centre Jülich, Institute of Neuroscience and Medicine (INM-1), Jülich, Germany; 8 University of Social Sciences and Humanities, Warsaw, Poland

**Keywords:** VBM, Dyslexia, Cognitive deficits, Heterogeneity

## Abstract

The variety of different causal theories together with inconsistencies about the anatomical brain markers emphasize the heterogeneity of developmental dyslexia. Attempts were made to test on a behavioral level the existence of subtypes of dyslexia showing distinguishable cognitive deficits. Importantly, no research was directly devoted to the investigation of structural brain correlates of these subtypes. Here, for the first time, we applied voxel-based morphometry (VBM) to study grey matter volume (GMV) differences in a relatively large sample (*n* = 46) of dyslexic children split into three subtypes based on the cognitive deficits: phonological, rapid naming, magnocellular/dorsal, and auditory attention shifting. VBM revealed GMV clusters specific for each studied group including areas of left inferior frontal gyrus, cerebellum, right putamen, and bilateral parietal cortex. In addition, using discriminant analysis on these clusters 79 % of cross-validated cases were correctly re-classified into four groups (controls vs. three subtypes). Current results indicate that dyslexia may result from distinct cognitive impairments characterized by distinguishable anatomical markers.

**Electronic supplementary material:**

The online version of this article (doi:10.1007/s00429-013-0595-6) contains supplementary material, which is available to authorized users.

## Introduction

Developmental dyslexia is defined as a specific deficit in reading acquisition that cannot be accounted for by low intelligence, poor educational opportunities, or any obvious sensory or neurological damage. Numerous theories were proposed trying to identify potential causes of dyslexia, however, no consensus was reached yet with regard to the neurological and cognitive basis of the disorder. It can be partly explained by the fact that the large body of data on cognitive deficits in dyslexia fails to fit into a single coherent theoretical framework and partly by the fact that the disorder is heterogeneous (Ramus and Ahissar 2012).

There is great variability in how dyslexia can be expressed in an individual relative to another depending on particular cognitive deficits that are present or not. Attempts were made to find distinguishable subtypes of the disorder. Several studies examined different dyslexia theories in multiple case studies of either adults (Ramus et al. 2003) or children (White et al. 2006) trying to assess the prevalence of each of the studied cognitive deficit adopting a criterion of deviance = 1.65 SD. In both aforementioned studies, performed on British native speakers, the phonological deficit was the most common, while the sensory deficits (magnocellular, auditory, motor/cerebellar) were less represented. Another study using a different criterion for deviance (below the 10th percentile) tried to determine if a proportion of dyslexic children could be characterized as suffering mainly from a selective visual span deficit or a phonological impairment (Bosse et al. 2007). The research showed that both French and British dyslexic children could be divided into four subgroups—having selective phonological, selective visual span deficit, both or none. Finally, using cluster analysis, Heim et al. (2008) divided German dyslexic children into three clusters with different cognitive deficits. Specifically, cluster no. 1 compared to age-matched controls had worse phonological awareness, cluster no. 2 had an attention deficit, whereas cluster no. 3 performed worse on phonological, auditory, and magnocellular tasks.

These studies show that distinguishable phenotypes of dyslexia exist on the cognitive level. There is, however, much more limited understanding of the potential neural markers of these specific subtypes. At the neurofunctional level, first attempts were made to relate cognitive profiles of dyslexia to brain activation during reading (Heim et al. 2010b), phonological awareness, visual-spatial attention, visual processing, and auditory processing (Heim et al. 2010a; for a review see Heim and Grande 2012). However, we are not aware of any study successfully defining subtypes of dyslexia and providing evidence for distinct cognitive and neuroanatomical profiles associated with each subtype.

Previous voxel-based morphometry (VBM) studies revealed differences in brain structure between children and adults with developmental dyslexia and controls. When summarized in a review (Richardson and Price 2009), increases and decreases of grey matter volume (GMV) in group comparisons of developmental dyslexics and good readers, constitute a widely distributed set of regions in both left and right hemispheres. The most frequently reported areas include: posterior temporal/temporo-parietal regions with increases and decreases of GMV (Brambati et al. 2004; Hoeft et al. 2007; Silani et al. 2005; Steinbrink et al. 2008), decreases of GMV in the left inferior frontal (Brown et al. 2001; Eckert et al. 2005), decreases of GMV in the occipito-temporal regions bilaterally (Eckert et al. 2005; Brambati et al. 2004; Kronbichler et al. 2008), and decreases of GMV in the cerebellum bilaterally (Brown et al. 2001; Brambati et al. 2004; Eckert et al. 2005; Kronbichler et al. 2008). Finally, there are also studies where no differences in GMV between dyslexic and control groups were revealed (Pernet et al. 2009). Taking into account the heterogeneity of behavioral deficits, it is not surprising that VBM studies of dyslexia do not always agree one with the other, depending on the sample at hand, its age and the behavioral profile.

Here, we aimed at distinguishing specific dyslexic subtypes based on examining four cognitive domains: phonological awareness, rapid automatized naming, visual magnocellular-dorsal processing, and auditory attention shifting. We hypothesize that dyslexic subtypes can be characterized by a specific pattern of GMV.

## Method

### Subjects

Eighty-one Polish native speakers took part in the study: 46 dyslexic children (mean = 123.1 months, SD = 10.6, 20 girls) and 35 control children (mean = 124 months, SD = 10.6, 22 girls). They had normal IQ (>85), were born at term (>37 weeks), were right-handed, attended school regularly, and none of them had any history of neurological illness or brain damage and no symptoms of ADHD. The groups were also matched for parental socioeconomic status (SES). Dyslexic children were recruited from education authorities and dyslexia centers and most of them had received some degree of remedial instruction in reading, spelling or oral language. The study was approved by the Warsaw Medical University Ethical Committee and all children and their parents gave written informed consent to the study.

### Procedure

Testing was performed in three successive phases: (i) behavioral tests of reading and writing, (ii) Wechsler intelligence subtests (WISC-R) and other cognitive functions described in details below, and (iii) an MRI scan.

### Behavioral tests

#### Reading tests

Reading ability was assessed using real word reading (WDREAD) from the normalized Polish battery of tests used for diagnosis of dyslexia (Bogdanowicz et al. 2008). Participants had to read aloud single words as quickly and accurately as possible. A measure of correctly read words per second was used. The majority of dyslexic children had independently received a diagnosis of developmental dyslexia. Non-word reading (NWREAD) was taken from the same battery. Children had to read aloud pseudowords as quickly and accurately as possible and a measure of correctly read pseudowords per second was used. Spelling was measured as percent of correctly typed words from a dictation of text.

#### Phonological awareness

Phonological awareness was assessed using phoneme deletion task (PHONDEL) (Bogdanowicz et al. 2008). In each item, a child has to delete a given phoneme from the spoken word, either at the beginning, in the middle or at the end of the word. The percent of accurately produced items was recorded.

#### Rapid automatized naming

Rapid automatized naming (RAN) was assessed using two non-alphabetic subtests of a Polish version of the test, i.e., objects and colors. A standardized score for time needed for naming both objects and colors was used.

#### Magnocellular-dorsal functions

Coherence motion thresholds (CMT) were measured using a random dot kinematogram consisting of a patch of 300 white dots (0.05°) that were randomly distributed within a 12° × 12° square on a black background (viewing distance 57 cm) on a 15.5″ screen. A variable proportion of these dots moved coherently, at a velocity of 15°/s, either upwards or downwards amongst the remaining randomly moving dots (Brownian motion). Stimuli were presented for 3 s, with each animation frame lasting 25 ms. The dots had a lifetime of 150 ms after which they reappeared in a random position. Subjects reported the direction of perceived coherent motion by pressing an appropriate mouse key. The threshold was determined by a 3 dB-up, 1 dB-down, two alternative forced-choice staircase procedure. Threshold was computed by taking the geometric average of the last 8 of 10 reversal points. Each series was repeated three times and the mean of two best series comprised individual’s overall motion coherence threshold (Talcott et al. 2000).

#### Auditory attention shifting

Auditory stream segregation threshold (SST) was employed following the Lallier et al. (2009) study. The auditory sequences were composed of high (1,000 Hz) and low (400 Hz) pitch pure tones presented in alternation. Each sequence lasted 5 s. Tones lasted 40 ms (including 5 ms linear onset/offset amplitude ramps). Stimuli were presented binaurally through headphones (at approximately 65 dB). Within each trial, a fixation cross, subtending 0.5° × 0.5° of visual angle appeared at the center of the screen followed by the auditory sequence after 500 ms. Children reported in a forced-choice paradigm whether they had perceived one stream or two streams. The threshold was determined by a ‘one-up, one-down’ adaptive two forced-choice method. Each sequence of alternating tones depending on stimulus onset asynchrony (SOA) leads to either a one- (connected) or a two-stream (segregated) percept. On the basis of the subject’s response, the computer program automatically either shortened the SOA (after ‘connected’ answer) or lengthened it. The session included 30 sequences and started with a 300 ms SOA. The SOA was first decreased or increased by steps of 40 ms and by steps of 20 ms after the first categorical change, then by steps of 10 ms after the second categorical change, and, finally, by steps of 5 ms after the third categorical change. The SST was defined as the mean SOA over the last 10 trials. This measure corresponded to the SOA at which participants could no longer dissociate the one-stream from the two-stream percepts and reflected the highest speed at which participants were able to shift automatically their attentional focus.

### Behavioral data analysis

All analyses were conducted with SPSS 18 (SPSS Inc., Chicago, IL, USA). Following Heim et al. (2008, 2010a), the existence of subtypes within the dyslexic sample was tested using a two-step cluster analysis. A detailed description of the procedure together with the parameters can be found in Heim et al. (2008). Here, the PHONDEL scores, the RAN, the CMT, and the SST were entered as variables of interest. Reading and IQ were not included since they served as diagnostic inclusion criteria. Next, the resulting three clusters were compared to the control group and each other with respect to reading ability and cognitive measures using one-way ANOVA followed by pairwise comparisons with Bonferroni correction applied.

### MRI data acquisition

Imaging data was acquired using 1.5-Tesla MRI scanner (Magnetom Avanto; Siemens, Erlangen, Germany) equipped with 32-channel phased array head coil. Detailed anatomical data of the brain were acquired with sagittal T1-weighted (time repetition = 1,720 ms; time echo = 2.92 ms) and T2-weighted (TR = 3,200 ms; TE = 381 ms) MPRAGE sequences with isotropic voxel size (1 × 1 × 1 mm).

### VBM analyses and statistics

Statistical Parametric Mapping 8 (Wellcome Trust Center for Neuroimaging, London, UK) was used for data processing and statistical analyses. New segment algorithm was applied in order to obtain basic tissue classes (Ashburner and Friston 2005) with pediatric priors created using Template-O-Matic toolbox (Wilke et al. 2008). Next, a study specific template was obtained using the Diffeomorphic Anatomical Registration Through Exponentiated Lie Algebra (DARTEL) toolbox (Ashburner 2007). Finally, images were spatially normalized to MNI space, modulated and smoothed with 6-mm isotropic Gaussian kernel.

Regional differences in GMV between controls and three dyslexic clusters were calculated using one-way ANOVA with age, sex, SES, and total intracranial volume as nuisance variables. Clusters from whole brain exploratory analysis (*p* < 0.001) were corrected using non-stationary cluster extended correction (*p* < 0.05) as implemented in VBM8 toolbox, which is crucial to adjust cluster sizes according to local roughness (Hayasaka et al. 2004).

In order to characterize GMV for each group, we performed *t* tests comparing each group against all others. This analysis was meant to reveal the most characteristic GMV features for each group. Then, to dissociate unique and shared GMV effects for different groups we performed additional contrasts using inclusive masking option in SPM. Contrasts to be masked were maintained at *p* < 0.001, whereas the inclusive mask was thresholded at *p* < 0.05 as implemented in previous studies (Uncapher and Rugg 2009; Joly et al. 2012).

Next, average GMV signal from all the significant clusters in the first analysis was extracted using the MarsBaR toolbox (Brett et al. 2002). Then the GMV of significant clusters was fed as predictor into a discriminant analysis in order to test how well the diagnostic group membership would be predicted by the GMV values. The independent variables (i.e., the GMV values per region) were entered together. The prior probabilities were adapted to the group sizes. The analysis was based on Wilk’s lambda. In order to assess the re-classification of the subjects into the diagnostic groups based on their GMV values, we used the leave-one-out classification approach, which is more conservative than the standard version because it eliminates the influence of each classified data-point on the sample to which it is compared.

## Results

### Behavioral data

As shown in Table 1, the dyslexic and control children differed significantly in WDREAD and NWREAD, spelling, phonological deletion (PHONDEL), and RAN. However, they did not differ significantly in case of CMT or SST.

**Table 1:** Differences in behavioral measures between control and dyslexic children

Measure	Control, mean (SD)	Dyslexic, mean (SD)	*t* test	*p*
WDREAD	1.02 (0.35)	0.29 (0.19)	10.93	<0.001
NWREAD	0.55 (0.14)	0.32 (0.14)	7.06	<0.001
Spelling	6.89 (1.45)	3.21 (1.35)	11.74	<0.001
PHONDEL	95.40 (5.05)	83.84 (13.68)	5.28	<0.001
RAN	5.89 (1.79)	4.20 (2.07)	3.85	<0.001
CMT	0.21 (0.05)	0.23 (0.08)	−1.01	ns
SST	160.93 (50.20)	183.15 (59.53)	−1.78	ns

*WDREAD* word reading, *NWREAD* non-word reading, *PHONDEL* phonological deletion, *RAN* rapid automatized naming, *CMT* coherent motion threshold, *SST* stream segregation threshold

### Cluster analysis

**Fig. 1:**
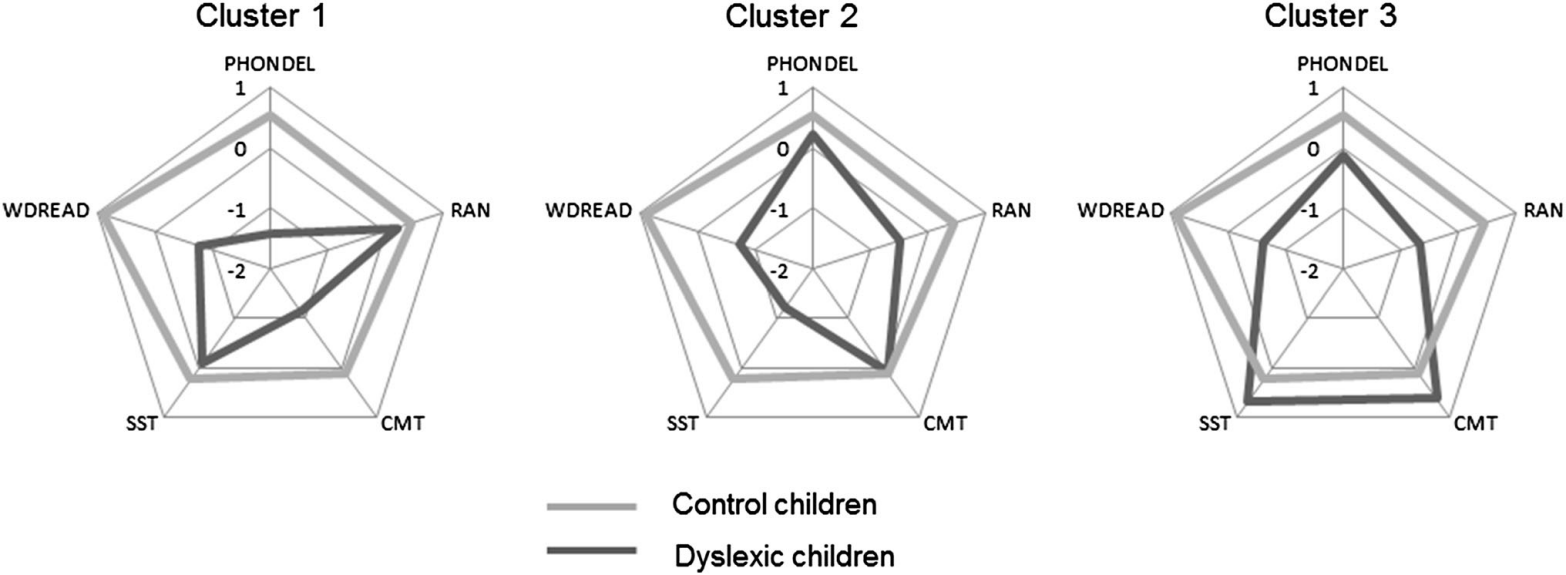
Comparison of behavioral scores in three clusters of dyslexic children against controls. For the visualization purposes, performance in each test was converted into *z* scores so that the positive values reflect better performance (for CMT and SST −*z* is presented)

The two-step cluster analysis for all four cognitive variables revealed three distinguishable clusters within the dyslexic children (see Fig. 1 for polar plots, bar plots are presented in Fig. S1). Cluster 1 had 14 subjects (30.4 %, 5 girls), cluster 2 had 15 subjects (32.6 %, 9 girls), and cluster 3 had 17 subjects (37 %, 6 girls). Groups did not differ significantly in age or SES.

For all four cognitive variables, the effect of group was significant: PHONDEL—*F*(3,77) = 25.14; *p* < 0.001; RAN—*F*(3,77) = 7.93; *p* < 0.001; CMT—*F*(3,77) = 12.01; *p* < 0.001; SST—*F*(3,77) = 18.01; *p* < 0.001 (see Table 2). In case of PHONDEL, dyslexic cluster 1 (Dys 1) performed the worse of all groups (for all comparisons *p* < 0.001), cluster 3 (Dys 3) performed worse than controls (*p* = 0.015), while cluster 2 (Dys 2) did not differ from controls. In RAN, Dys 2 and 3 had lower scores than controls (*p* = 0.005 and *p* < 0.001, respectively). In CMT, the highest threshold of all groups had Dys 1 (all *p* < 0.001), whereas in SST the highest threshold of all groups had Dys 2 (all *p* < 0.001). All three clusters performed significantly worse than controls on WDREAD, NWREAD, and spelling (all *p* < 0.001), while there were no differences between the dyslexic clusters.

**Table 2:** Differences in behavioral measures between control group and dyslexic clusters

Measure	Controls, mean (SD)	Cluster 1, mean (SD)	Cluster 2, mean (SD)	Cluster 3, mean (SD)	*F*	*p*	Post hoc *p* < 0.05
WDREAD	1.02 (0.35)	0.26 (0.19)	0.28 (0.19)	0.33 (0.21)	45.51	<0.001	CON > 1, 2, 3
NWREAD	0.55 (0.14)	0.33 (0.13)	0.30 (0.15)	0.34 (0.15)	16.67	<0.001	CON > 1, 2, 3
Spelling	6.89 (1.45)	2.86 (0.95)	3.07 (1.22)	3.53 (1.50)	52.17	<0.001	CON > 1, 2, 3
PHONDEL	95.40 (5.05)	71.43 (15.32)	91.59 (7.25)	87.21 (9.28)	25.14	<0.001	CON > 1, 3
							2, 3, CON > 1
RAN	5.89 (1.79)	5.36 (1.78)	3.87 (2.2)	3.53 (1.87)	7.93	<0.001	CON > 2, 3
CMT	0.21 (0.05)	0.30 (0.07)	0.21 (0.07)	0.17 (0.06)	12.01	<0.001	1 > 2, 3, CON
SST	160.93 (50.20)	178.75 (43.47)	243.07 (35.97)	133.91 (36.62)	18.01	<0.001	2 > 1 > 3
							2 > 1, 3, CON

*WDREAD* word reading, *NWREAD* non-word reading, *PHONDEL* phonological deletion, *RAN* rapid automatized naming, *CMT* coherent motion threshold, *SST* stream segregation threshold

### VBM results

The dyslexic clusters and control children did not differ significantly in the global brain measures, i.e., total intracranial volume, total grey matter, or white matter volume.

**Fig. 2:**
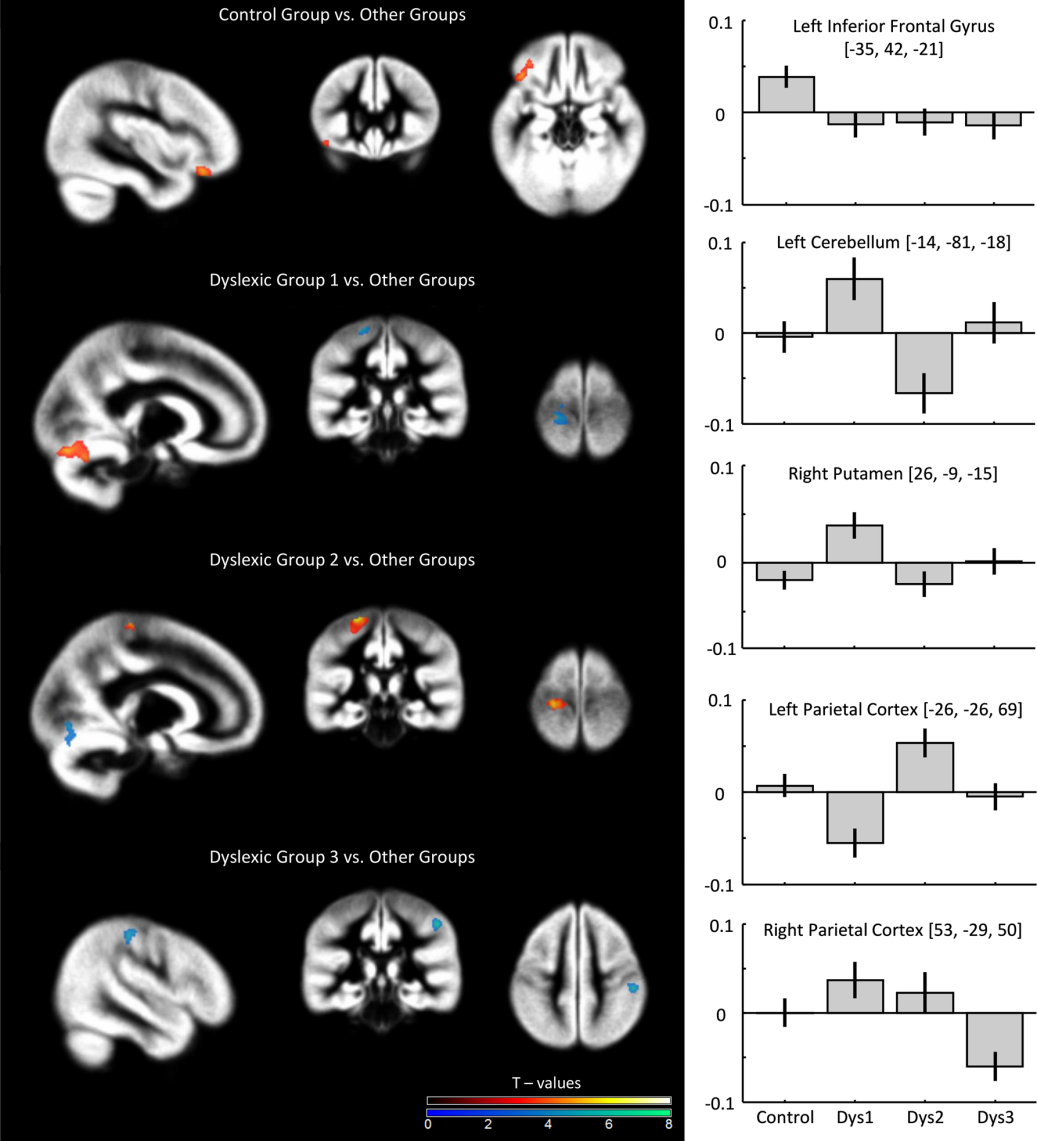
Grey matter volume differences between the groups (*in* *red* decreased GMV; *in* *blue* increased GMV) together with contrast estimates for five significant clusters. Results are displayed at uncorrected *p* < 0.001

**Fig. 3:**
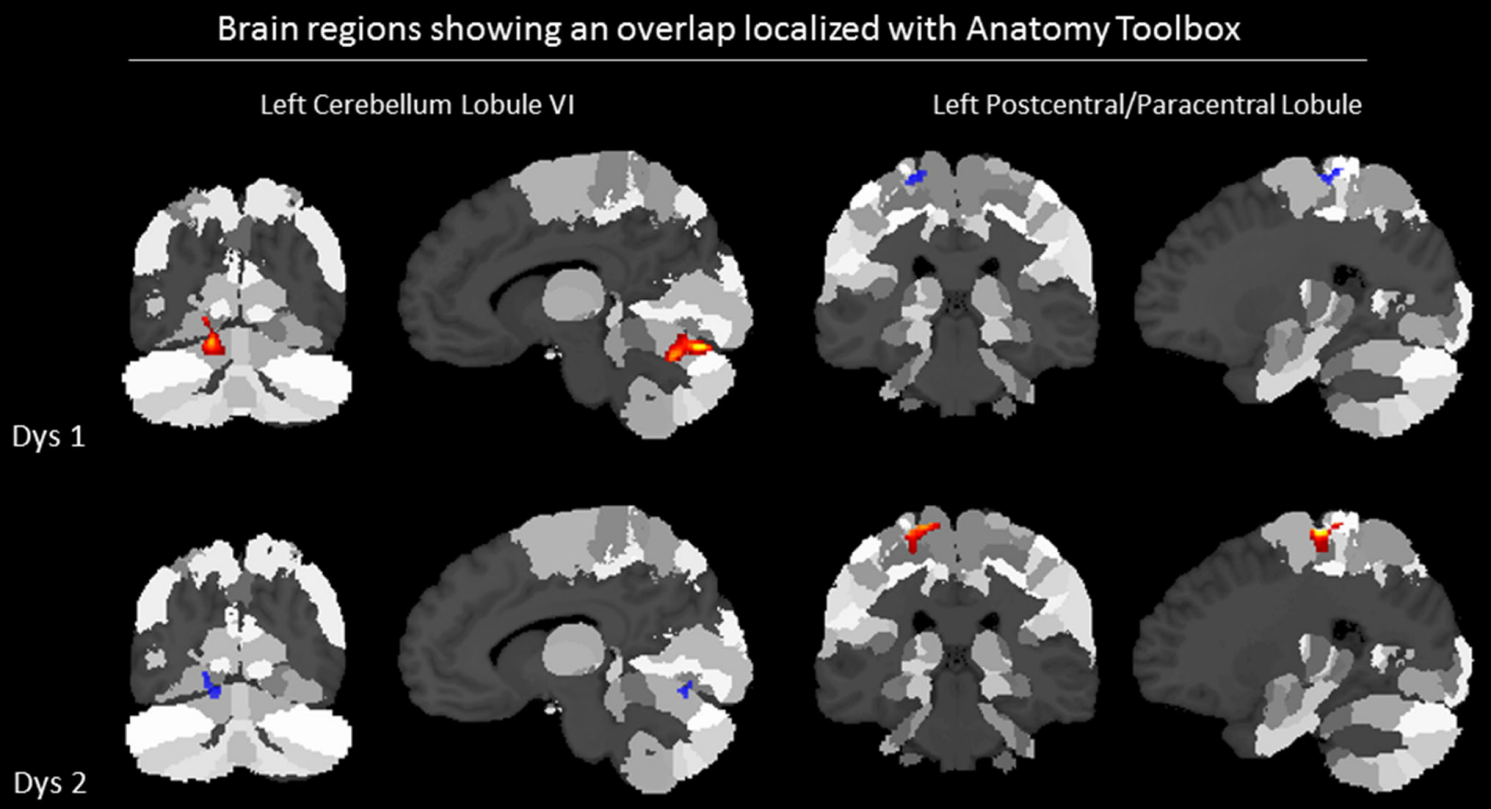
Brain regions displaying an overlap between the two dyslexic subgroups localized with Anatomy Toolbox (*in* *red* decreased GMV; *in* *blue* increased GMV)

With regard to the local GMV differences, VBM contrasts for controls versus all dyslexic subtypes revealed significantly reduced GMV for dyslexics compared to controls in the left inferior frontal gyrus (Table 3; Fig. 2). This structure was also revealed when each dyslexic subtype was contrasted to controls (using inclusive masking procedure) showing a common GMV reduction (Table 4). There were no significant differences in GMV for the reverse contrast (dyslexics > controls). When Dys 1 was compared to all other groups, a significant increase in GMV was revealed in the left cerebellum and the right putamen, while a significant reduction was observed in the right dorsal premotor cortex and the left parietal cortex. The increase in the left cerebellum and the right putamen together with a decrease in the right premotor cortex were revealed as unique to the first dyslexic subtype. Dys 2 compared to all other groups was characterized of higher GMV in the left parietal cortex (in a region overlapping with the one showing a decrease in Dys 1 group—see Fig. 3) and medial part of the right superior frontal gyrus, whereas lower GMV was found in left cerebellum (in a region overlapping with the one showing an increase in Dys 1 group). However, no unique effects were found for this subtype using inclusive masking procedure. Finally, Dys 3 compared to all other groups was characterized only by decreased GMV in the right parietal cortex, however, again no unique decreases or increases of GMV for this subtype were revealed. Additionally, there was a common decrease of GMV in the right anterior and middle cingulate gyrus in the first and second dyslexic subtype, whereas a common increase of GMV for the first and third dyslexic subtype was revealed in the left cerebellum.

**Table 3:** Brain regions with significant grey matter volume difference between the groups

Brain region	MNI coordinates			T-stat	Cluster size
	*x*	*y*	*z*		
Controls > all other groups					
L inferior frontal gyrus	−35	42	−21	4.42	392
All other groups > controls	–				
Dys 1 > all other groups					
L cerebellum, lingual gyrus	−14	−81	−18	4.97	529
R putamen	26	−9	−15	4.33	199
All other groups > Dys 1					
R dorsal premotor cortex (precentral, middle and inferior forntal gyri)	54	13	38	5.53	221
L parietal cortex (paracentral lobule and postcentral gyrus)	−24	−35	72	5.07	133
Dys 2 > all other groups					
L parietal cortex (paracentral lobule and postcentral gyrus)	−26	−26	69	6.14	352
R superior medial frontal gyrus	9	49	32	5.26	77
All other groups > Dys 2					
L cerebellum, lingual gyrus	−15	−77	−6	4.76	102
Dys 3 > all other groups	–				
All other groups > Dys 3					
R parietal cortex (supramarginal and postcentral gyri)	53	−29	50	6.11	125

Cluster size indicates number of voxels

*L* left, *R* right hemisphere

**Table 4:** Unique and shared grey matter effects for the different groups

Contrast	Brain region	MNI			T-stat	Cluster size
		*x*	*y*	*z*		
**Common decreased GMV for each dyslexic group**						
Control > all dys groups masked incl. (con > Dys 1 & con > Dys 2 & con > Dys 3)	L inferior frontal gyrus	−35	42	−21	4.42	276
**Unique decreases in GMV for Dys 1**						
Dys 1 < control masked incl. (Dys 1 < Dys 2 & Dys 1 < Dys 3)	R dorsal premotor (precentral, middle and inferior frontal gyri)	55	13	39	5.30	248
**Unique increases in GMV for Dys 1**						
Dys 1 > control masked incl. (Dys 1 > Dys 2 & Dys 1 > Dys 3)	R putamen	23	−3	−6	4.72	239
	L cerebellum (crus 1, VI), lingual gyrus	−14	−81	−19	4.70	399
**Unique decreases in GMV for Dys 2**		–				
**Unique increases in GMV for Dys 2**		–				
**Unique decreases in GMV for Dys 3**		–				
**Unique increases in GMV for Dys 3**		–				
**Common decreases in GMV for Dys 1 and Dys 2**						
Dys 1 & Dys 2 < control masked incl. (Dys 1 < con & Dys 2 < con & Dys 1 < Dys 3 &Dys 2 < Dys 3)	R anterior/middle cingulate gyrus	5	7	30	4.41	367
**Common increases in GMV for Dys 1 and Dys 2**		–				
**Common decreases in GMV for Dys 1 and Dys 2**		–				
**Common increases in GMV for Dys 1 and Dys 3**						
Dys 1 & Dys 3 > control masked incl. (Dys 1 > con & Dys 2 > con & Dys 1 > Dys 2 & Dys 3 > Dys 2)	L cerebellum (VI, crus 1), lingual gyrus	−21	−66	−19	4.18	864
**Common decreases in GMV for Dys 2 and Dys 3**		–				
**Common increases in GMV for Dys 2 and Dys 3**		–				

Cluster size indicates number of voxels. Contrasts to be masked were maintained at *p* < 0.001, whereas the inclusive mask was thresholded at *p* < 0.05

*L* left, *R* right hemisphere

Next, to test the re-classification of subjects into groups (controls versus three dyslexic subtypes) based on their GMVs, values from structures revealed by VBM analyses were fed into a discriminant analysis. GMV of seven structures (depicted in Table 5) served as independent variables, whereas the dependent variable was group membership. While the first clustering based on behavioral data was performed on dyslexics only, here assignment to one of four groups was possible. In the discriminant analysis, we used leave one out classification where each case in the analysis is classified by the functions derived from all cases other than that case. This analysis revealed three discriminant functions with 79 % of cross-validated grouped cases correctly classified. The accuracies for each group were as following: controls (85.7 %), Dys 1 (85.7 %), Dys 2 (60 %), Dys 3 (76.5 %).

**Table 5:** Correlations between discriminating variables and standardized canonical discriminant functions

Discriminating variables	Function		
	1	2	3
L parietal cortex	0.617^a^	0.219	−0.117
L cerebellum	−0.497^a^	−0.149	−0.031
R superior frontal gyrus	0.442^a^	0.318	−0.272
R putamen	−0.426^a^	0.027	−0.345
R dorsal premotor cortex	0.415^a^	−0.410	0.302
R parietal cortex	−0.085	0.788^a^	0.102
L inferior frontal gyrus	0.062	−0.017	0.825^a^

^a^Largest absolute correlation between each variable and any discriminant function

In addition, a validation of the GMV discriminant scores was accomplished by Pearson correlation analyses between the GMV discriminant scores of three functions and the behavioral variables (Table 5). Significant correlation was noted between the first discriminant function and PHONDEL (*r* = 0.38, *p* = 0.001). The second discriminant function was correlated with the two perceptual thresholds—CMT (*r* = 0.31, *p* = 0.005) and SST (*r* = 0.33, *p* = 0.003). Whereas, the third discriminant function was correlated with reading scores—WDREAD (*r* = 0.40, *p* < 0.001), NWREAD (*r* = 0.24, *p* = 0.029), spelling (*r* = 0.40, *p* < 0.001), and RAN (*r* = 0.29, *p* = 0.009).

## Discussion

In the present study, we identified three subtypes of dyslexia with distinct cognitive and neurobiological profiles. All had severe reading and spelling impairments compared to controls but did not differ in these measures with each other. On the neuronal level, they showed reduced GMV in the left inferior frontal gyrus relative to age-matched good readers consistent with previous studies (Brown et al. 2001; Eckert et al. 2005; Vinckenbosch et al. 2005). Importantly, this effect was common across all dyslexic subtypes. The left inferior frontal gyrus contributed mostly to the third discriminant function, which was associated with single word and pseudoword reading, spelling, and rapid naming. In line, the activity in this area increased with reading ability and was related to rapid naming (Turkeltaub et al. 2003). It was also shown that the size of inferior frontal gyrus, particularly pars triangularis can predict rapid naming speed in a group of children with predominating double deficit (Eckert et al. 2005).

The first subtype of dyslexic children had worse phonological awareness and magnocellular-dorsal skills compared to all other groups. A similar cluster was previously described by Heim et al. (2008) though in that study children besides phonological and magnocellular had also an auditory deficit. VBM revealed that compared to other groups this subtype was characterized of increased GMV in the left cerebellum, lingual gyrus and right putamen together with a decrease of GMV in the left parietal (mainly somatosensory) and right dorsal premotor cortices. However, only the differences in the left cerebellum, the right putamen and right dorsal premotor cortex were unique for this dyslexic subtype. The second dyslexic subtype had a reverse cognitive profile compared to first subtype, i.e., while phonological and magnocellular-dorsal skills were comparable to controls, the children showed impairments in rapid naming and auditory attention shifting. This was nicely reflected in GMV profiles since in the second subtype a decrease in GMV in the left cerebellum, lingual gyrus and an increase of GMV in the left parietal (somatosensory) cortex were observed, in regions overlapping with the ones showing a reverse pattern in subtype 1, together with an increase of GMV in the medial part of the right superior frontal gyrus. However, no unique effects were revealed for this subtype.

The structures differentiating subtypes 1 and 2 contributed to the first discriminant function, which was associated with phonological awareness. Previous anatomical studies yielded inconsistent results for the cerebellum of dyslexic subjects showing either decreased GMV compared to controls (Brown et al. 2001; Brambati et al. 2004; Eckert et al. 2005; Kronbichler et al. 2008) or no differences between the groups followed by a negative association between cerebellar GMV and phonological skills in controls (Kibby et al. 2008; Pernet et al. 2009). Nicolson et al. (2001) have proposed two mechanisms by which cerebellum may play a role in dyslexia. The first is associated with so-called motor-articulatory feedback hypothesis (Heilman et al. 1996), which suggests that recognition of phonemes is dependent on awareness of the positions and movements of the articulatory system. Poor quality articulatory representations lead to impaired sensitivity to the phonemic structure of language and to reduced phonological awareness. The second is related to decreased processing speed, which is reflected in difficulties with rapid naming.

Left somatosensory and right premotor cortices GMV reduction in the first subtype might suggest problems with articulatory feedback, which produces a severe phonological deficit. This in turn might cause greater reliance on silent articulatory processes (Wimmer et al. 2010) when dealing with decoding resulting in increased GMV in cerebellum and putamen. The role of the latter in reading, mainly silent articulation (Hernandez and Fiebach 2006) and phonology (Tettamanti et al. 2005) has been shown previously although it was not specifically linked with dyslexia.

Interestingly, one structure, the right anterior/middle cingulate gyrus showed a common decrease of GMV for both the first and second dyslexic subtype. This region is widely believed to play a role in cognitive control, helping to resolve conflict from distracting events by focusing attention towards task-relevant stimuli (Weissman et al. 2005). Several studies have suggested a role of anterior cingulate during anticipation (Murtha et al. 1996) and voluntary attentional orienting (Hopfinger et al. 2000; Weissman et al. 2002). It was also shown that activity in this region was correlated with the level of attention dedicated to learning events (Bryden et al. 2011). Both the first and the second dyslexic subtype, although at first glance seemingly different with regard to the behavioral profile, showed deficits in two different tasks requiring high amounts of attentional control—coherent dot motion and stream segregation. It was shown that performance in the formed is largely modulated by attention orienting (Liu et al. 2006), whereas the latter is regarded as a measure of attention shifting (Lallier et al. 2009). It seems possible that the reduced GMV in the right anterior cingulate might lead to poorer performance on these two different tasks involving attention focusing. In line, anterior cingulate was consistently revealed as having decreased GMV in attention deficit disorder (Amico et al. 2011; Seidman et al. 2006). It remains, however, unclear why these two dyslexic subtypes are impaired either in coherent motion or SST and not in both. Most probably, the whole pattern of GMV changes and not only differences the right anterior cingulate influence the behavioral outcome.

The third subtype had a double deficit [described before by Wolf and Bowers (1999)], whereas it had preserved magnocellular-dorsal and attentional shifting skills. Compared to other groups it was characterized of lower GMV in the right parietal cortex, however, no unique effects were found for this subtype. The right parietal cortex contributed to the second discriminant function significantly correlated with both perceptual thresholds—magnocellular-dorsal and auditory attention shifting. The role of right inferior parietal cortex for attention was well documented (Behrmann et al. 2004). It is also known that visual input the right parietal cortex projects from the magnocellular layers of the lateral geniculate nucleus (Eden and Zeffiro 1998). We found that the lower the perceptual thresholds the lower the GMV in the right inferior parietal cortex, in agreement with previous studies showing significantly larger GMV in inferior parietal lobule in adults with ADHD (Seidman et al. 2011). Additionally the third subtype showed common with the first subtype increase of GMV in the left cerebellum cluster, possibly reflecting deficient phonological awareness skills.

On the basis of the GMV in the significant clusters described above using a discriminant analysis, 79 % of cross-validated cases were correctly re-classified into four groups (controls versus three dyslexic subtypes).

In conclusion, our results are in line with the hypothesis that dyslexic subtypes can be characterized by specific patterns of GMV. We have dissociated three different groups of dyslexic behavior and identified brain areas with local GMV that differs from controls and between dyslexic groups. However, it seems that the relationship between brain structure and behavior is more complicated than anticipated, i.e., there is no clear, unique patterning of GMV differences. The obtained results form an intricate pattern of differences and not a plain 1:1 association between one subgroup and one region. Thus, based on a GMV difference in a specific brain area it is not possible to univocally predict the behavioral phenotype.

On the other hand, taking into account the complex aetiology of dyslexia it is not surprising that the array of GMV differences is also complex. Besides, having only four different cognitive tests, one cannot expect to fully describe the behavioral and neural phenotypes of dyslexia. Nevertheless, our study shows that it is important to look for potential subtyping of this disorder both on the behavioral and brain level. Further studies with a larger battery of cognitive tests and bigger sample size are needed to verify current findings. Lastly, it would be important to examine whether the revealed profiles (subtypes) are stable in development and whether they can be differentiated in younger children at the pre-reading stage.

## Electronic supplementary material

Below is the link to the electronic supplementary material.

Supplementary Fig. S1. The comparison of behavioral scores in three clusters of dyslexic children and controls. For the visualization purposes performance each test was converted into z-scores so that the positive values reflect better performance (for CMT and SST –z is presented). Error bars represent standard deviation. *p < 0.05; **p < 0.01; *** p < 0.001 (TIFF 40 kb)
