# Supplementary figures and images for: Cognitive subtypes of dyslexia are characterized by distinct patterns of grey matter volume
Source: Brain Struct Funct. 2013 Jun 18;219(5):1697–707. doi: 10.1007/s00429-013-0595-6 (PMC4147248; doi:10.1007/s00429-013-0595-6)

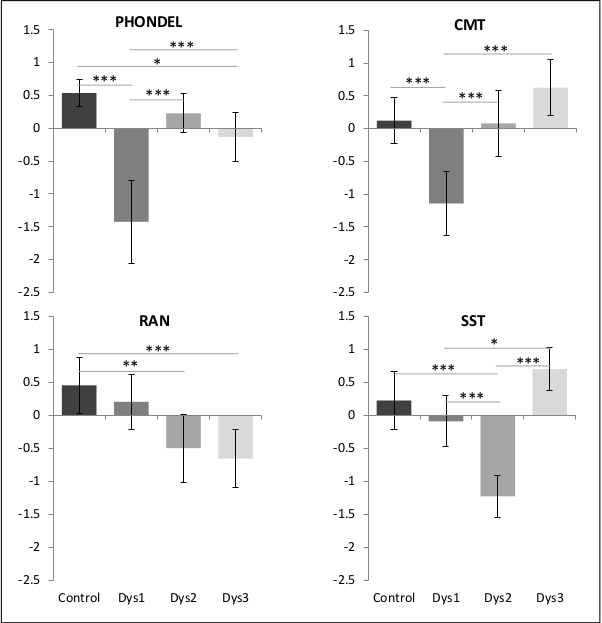

Supplement: Supplementary file 1 — Supplementary Fig. S1. The comparison of behavioral scores in three clusters of dyslexic children and controls. For the visualization purposes performance each test was converted into z-scores so that the positive values reflect better performance (for CMT and SST –z is presented). Error bars represent standard deviation. *p < 0.05; **p < 0.01; *** p < 0.001 (TIFF 40 kb) [file 429_2013_595_MOESM1_ESM.tif]
